# Processing Code-Switches in the Presence of Others: An ERP Study

**DOI:** 10.3389/fpsyg.2020.01288

**Published:** 2020-06-26

**Authors:** Edith Kaan, Souad Kheder, Ann Kreidler, Aleksandra Tomić, Jorge R. Valdés Kroff

**Affiliations:** ^1^Department of Linguistics, University of Florida, Gainesville, FL, United States; ^2^School of Communication Science and Disorders, Florida State University, Tallahassee, FL, United States; ^3^Department of Spanish and Portuguese Studies, University of Florida, Gainesville, FL, United States

**Keywords:** code-switching, early frontal positivity, LPC, social factors, bilingual language processing, pro-active control, language control

## Abstract

Code-switching is highly socially constrained. For instance, code-switching is only felicitous when those present are fluent in both languages. This means that bilinguals need to dynamically adjust their language control and expectation of code-switching to the current social situation or context. The aim of the present EEG study was to investigate how and when language control in the comprehension of code-switches is affected by the assumed language knowledge of others in the context. Spanish-English bilinguals read sentences with and without code-switches together with another Spanish-English bilingual or with an English monolingual. Switches elicited an early fronto-central positivity. This effect was smaller overall when a bilingual was present at the start of the study. In addition, the late positive complex found for switches was smaller when a bilingual was present rather than a monolingual, but only for those participants who were sensitive to the other’s language knowledge in their off-line judgments. These findings suggest that the bilinguals in our study expected and activated both languages when initially paired with a bilingual and that they more easily accommodated code-switches, in the presence of a bilingual than in the presence of a monolingual. Our findings support the view that language control can be modulated by the perceived language knowledge of others present, and are compatible with a dynamic control model of bilingual language comprehension.

## Introduction

Bilinguals are faced with multiple linguistic options in their daily language use: which language is currently in use, or should be used? Is it appropriate to use only one language or to change between languages (code-switching)? Most of these choices are socially and pragmatically driven. For instance, bilingual communities differ in the extent to which code-switching is socially accepted (Poplack, 1988; Zentella, 1997; Bullock and Toribio, 2009; Gardner-Chloros, 2009; Torres Cacoullos and Travis, 2018). Furthermore, the use of code-switching is only felicitous when those present in the context are proficient in both languages and code-switch as well. This means that bilinguals need to dynamically adjust their language control to a dynamically changing social situation: in some cases one language needs to be selected and interference from the other avoided; in other cases both languages can be selected. Psycholinguistic models therefore need to specify how and when non-linguistic factors are used in language selection, inhibition or in switching between languages during production and comprehension. Current psycholinguistic research on code-switching and language switching has just recently taken social factors into account (Martin et al., 2016; Beatty-Martínez and Dussias, 2017; Blanco-Elorrieta and Pylkkänen, 2017; Kapiley and Mishra, 2018); however, no online study has investigated whether the co-presence of a mono- or bilingual affects the processing of code-switching.

Cognitive models of bilingual language control differ in how and when non-linguistic factors, such as the perceived language knowledge of others present, affect language control. The Control Process Model (CPM) proposed by Green and colleagues (Green and Wei, 2014; Green, 2018) is mainly a model of bilingual production. According to this model, the linguistic and non-linguistic context, including factors such as the speaker’s intention or attitude, control which items are let into the utterance planning process. Depending on the linguistic context and social situation, these items can be from one or both languages. Green and colleagues stress that language control is dynamically adjusted depending on the context. In a unilingual situation, one language can be active and the other language inhibited. Alternatively, one language can briefly cede control to the other, as in a situation in which a word from language A is inserted into Language B. In another situation, both languages are selected opportunistically and control is open, that is, not passed between the languages. This is the case when bilinguals code-switch many times within a sentence. Dynamic language control is associated with dynamic attentional control as well: unilingual situations are hypothesized to require a narrow focus of attention (focus on one language, inhibiting the other), whereas dense code-switching requires a broad attentional focus (coordinating both languages). In the CPM, the social context can be pro-actively taken into consideration: depending on who the bilingual is talking to, the items let into the production buffer can be from Language A, Language B, or either language.

It is unclear to what extent and how the presence of a bi- or monolingual interlocutor affects bilingual comprehension. Some current models of bilingual language comprehension such as the BIA+ model (Dijkstra and Van Heuven, 2002) and Multilink model (Dijkstra et al., 2019b) distinguish between lexicon-internal activation and task/decision processes. When a bilingual is reading or listening, lexical representations in both languages become active. Some representations are more active than others based on linguistic factors such as their frequency, language dominance, or the nature of the preceding words. For instance, if the conversation has been in English only, English words are more strongly activated than Spanish in a Spanish-English bilingual lexicon. When the next word is Spanish, it will take a while before the Spanish word becomes activated, leading to a “switch cost” (Chauncey et al., 2008). Task/decision processes operate on the activation in the lexicon, but do not change it (Dijkstra et al., 2019a). If the non-linguistic context, in our case, the perceived language knowledge of a partner, mainly affects the task/decision system, one would expect that activation in the lexicon will not be affected by what one assumes about the other’s language background. On the other hand, in a model in which task/decision factors can affect lexical activation, e.g., by means of a “language node” (as in the BIA model, Dijkstra and Van Heuven, 1998), such non-linguistic factors can affect the lexicon-internal activation and may modulate activation levels in advance of the linguistic input.

There is some evidence that the perceived language knowledge of others affects language activation and control. For instance, listeners typically show a P600 effect for syntactic errors versus their grammatical counterparts in the event-related potentials (ERP) when listening to native-accented speech. However, when presented with certain grammatical errors in second-language accented speech, listeners showed a smaller or no P600 (Hanulikova et al., 2012; Caffarra and Martin, 2018). Perceived L2 proficiency of a partner also affects language choice in naming tasks, such that speakers switched into the L2 less often when led to believe they were dealing with a less proficient L2 speaker (Kapiley and Mishra, 2018). Furthermore, bilingual listeners responded more slowly when a person introduced as a monolingual speaker produced items in the other language rather than the expected language (Molnar et al., 2015). Blanco-Elorrieta and Pylkkänen (2017) investigated magneto-encephalographic (MEG) responses to production and perception of cued language switches. In some conditions, cues were static portraits of people introduced as mono- or bilingual; in other conditions a color indicated which language was to be used. Switch effects were smaller or absent in the socially cued conditions than in conditions that used color as a switch cue. Martin et al. (2016) found differences in ERPs related to the onset of the video of a person introduced as being bilingual versus monolingual, even before the language input. In particular, the P3b, a component associated with context updating and goal activation (Polich, 2007), was larger at the onset of a video of a bilingual versus monolingual individual, even before they spoke. The N1 component (sensitive to lexicality) was larger for pseudowords versus real words spoken by a person introduced as monolingual in the video, but not for individuals introduced as bilingual, even though the words were of the same language in both conditions. Furthermore, the difference in N400 (indexing lexical processing) between pseudowords and real words was larger for words spoken by a monolingual than a bilingual. The latter effect correlated with the P3b effect at the video onset. These results suggest that the knowledge of somebody being bi- or monolingual pro-actively adjusts language control. This pro-active adjustment subsequently can affect the lexical processing of words presented later. This indicates that participants were neurally more efficient when detecting pseudowords versus real words when they knew the talker in the video was monolingual and could expect a particular language to be used.

In the present study we used Event-Related brain Potentials (ERPs) to investigate whether the co-presence of a monolingual or bilingual individual affects the processing of code-switching during comprehension, and if so, at what stage of processing. For practical purposes, we restrict ourselves to written sentence contexts. Studies investigating code-switching in sentences using self-paced reading typically find a switch-effect; that is, response times are longer at the point of the switch compared to non-switch controls. This switch effect is modulated by the direction of the switch as well as the reader’s language dominance (Bultena et al., 2015; Litcofsky and Van Hell, 2017). Electrophysiology, and in particular ERPs, allows one to more closely look at the timing and subprocesses involved in the processing of code-switches. Several ERP components have been found to be sensitive to code-switching in written sentence contexts (see for overviews Van Hell et al., 2015, 2018). One component that has been consistently reported for a code-switched versus a control word is the late positive component (LPC, 500–900 ms after onset, e.g., Moreno et al., 2002; Van Der Meij et al., 2010; Ng et al., 2014; Litcofsky and Van Hell, 2017), especially when the switch is into the non-dominant language (Litcofsky and Van Hell, 2017). In the context of code-switching, the LPC has been interpreted as sentence-level revision (Litcofsky and Van Hell, 2017) or unexpected events triggering stimulus evaluation and memory updating (Moreno et al., 2008). In addition, the LPC has been found to be modulated by social norms. For instance a larger LPC has been observed for recordings in which the content did not match the stereotypical representations of the gender of the recording voice (although the authors refer to this component as a P600 Lattner and Friederici, 2003), and for emotional or negative stimuli (Schupp et al., 2003, 2004; Schacht and Sommer, 2009; Fields and Kuperberg, 2012). Based on this, it is likely that the LPC for code-switches is affected by the degree to which the switch is (socially and emotionally) expected.

A second component of interest to our study is an early frontal positivity (around 200–300 ms). This component has been reported for a code-switched versus a control word in those participants who do not socially accept switching (Beatty-Martínez and Dussias, 2017). The early frontal positivity (P2 or P3a) has been found to be modulated by top-down attention in non-linguistic visual selection tasks (Luck and Hillyard, 1994). In language comprehension studies, the early frontal positivity has been found to be larger when a word form is highly expected given the preceding context (although mainly when stimuli were presented to the right visual fields/left hemisphere, Federmeier and Kutas, 2005; Federmeier et al., 2005; Wlotko and Federmeier, 2007). This has been interpreted as the P2 reflecting more efficient extraction of visual information due to top-down expectations (Federmeier et al., 2005). In the context of code-switching, the early frontal positivity has been associated with shifts of attention from the expected to the unexpected language, or from a narrow to a broader focus of attention (Beatty-Martínez and Dussias, 2017). The P2 can therefore index pro-active control: if both languages are expected and selected, attention is already broad and would not need to shift from one to the other when a code-switch occurs. A small or no difference in the early frontal positivity for a code-switch versus a non-switch control may therefore suggest a pro-active selection of both languages.

Other components that have been found to be modulated by written code-switches are the N400 component (central distribution over the scalp), and a left anterior negativity (LAN), that is, a negativity with a more left anterior distribution (Moreno et al., 2002; Proverbio et al., 2004; Van Der Meij et al., 2010; Ng et al., 2014). These components occur around 300–500 ms after onset of the critical word, with the switch word eliciting a larger negative amplitude than the no-switch control words. The N400 has been mainly associated with semantic processing (Kutas and Federmeier, 2011); the LAN has been associated with working memory or morpho-syntactic processing (Coulson et al., 1998). If lexical processing can be affected by non-linguistic factors, one would also expect these components to be modulated by the presence of a bilingual or monolingual in the context.

The present study is inspired by the joined reading task used by Rüschemeyer et al. (2015). Rüschemeyer et al. (2015) had participants read sentences, some of which contained an ending that was semantically anomalous when the sentence was presented in isolation, such as *The boy had gills*. Sentences were preceded by a context sentence presented over headphones. In some conditions, this context made the target sentence plausible, e.g., *In the boy’s dreams, he could breathe under water*. When the context sentence was presented over headphones, and the participant was alone, no N400 effect was seen for *gills* versus a semantically plausible control sentence. However, if another person was present who could not hear the supporting context sentence that the participant heard, an N400 was elicited at *gills* in the participant’s ERPs, indicating that the participant took into account what the interlocutor knew despite the participant’s own privileged knowledge. This effect was dubbed the SOCIAL N400 effect. A follow-up study by Jouravlev et al. (2019) reports a social N400 effect only if the participant was asked to evaluate whether the sentence made sense, either for the other person present, or in general.

In the current study, we tested whether the co-presence of a bilingual or a monolingual affected the processing of code-switches. We presented Spanish-English bilinguals with sentences that either contained an English to Spanish code-switch or were in English only. In the main study, Experiment 2, the participant read the sentences jointly with another Spanish-English bilingual in one half of the study and an English monolingual in the other half (order counterbalanced). Experiment 1 was a control study in which the participant read sentences alone in the booth. The aim of this control study was to test the effect of code-switching in our specific materials and to see to what extent the effects were different between the first and second half of the study in the absence of any social context manipulation. Based on previous studies on written code-switches, we expected code-switches to elicit a frontal positivity, N400, LAN and/or LPC versus control words in unilingual sentences in both Experiments. If bilinguals can adjust their expectation of code-switching and use of the other language based on the assumed language knowledge of others, we expected these switch effects to be smaller in the presence of a bilingual than a monolingual in Experiment 2. Since in particular the early frontal positivity has been associated with shifts of attention from the expected to the unexpected language (Beatty-Martínez and Dussias, 2017), the reduction or absence of an early frontal positivity for switches in the presence of a bilingual versus monolingual would suggest that bilinguals pro-actively adjust the levels of activation of the languages in the presence of another bilingual.

## Experiment 1

### Materials and Methods

#### Participants

Sixteen right-handed, healthy young adult Spanish-English bilinguals participated in the study either for course credit or a US $10/h monetary compensation. Data from two more participants were collected but not included in the analysis because of technical failures (one participant) or many artifacts in the data (one participant who had fewer than 20 trials for at least one condition after artifact rejection). Participant characteristics are summarized in Table 1. Language dominance was judged by the performance on a modified Boston Naming Task (Kaplan et al., 1983) as used in e.g., Guzzardo Tamargo et al. (2016). Our participants correctly named more pictures in English than in Spanish, except for two participants who scored better in Spanish. Below we report the analysis including all participants. Since language dominance has been shown to affect the processing of code-switches, we also conducted analyses without these Spanish-dominant participants (Litcofsky and Van Hell, 2017). However, these analyses yielded the same effects as the analysis reported below (see Supplementary Material).

**TABLE 1:** Characteristics of the participant groups in Experiments 1 and 2.

	Exp. 1	Exp. 2 Monolingual first	Exp. 2 Bilingual first
N (gender)	16(2m,14f)	16(5m,11f)	17(2m,15f)
Age in years (range)	20.4(18−28)	19.9(18−28)	19.35(18−21)
AoA Spanish in years (range)	0.0(0−0)	0.5(0−8)	0.3(0−3)
AoA English in years (range)	3.6(0−6)	3.9(0−9)	3.0(0−9)
Frequency of using code-switching (5 = always)	3.63 (1.15)	3.31 (0.95)	3.82 (0.88)
Frequency of encountering code-switching (5 = always)	3.88 (0.62)	3.75 (0.93)	3.35(0.70)^∗^
MELICET	43.3 (4.5)	45.5 (2.4)	44.0 (3.76)
DELE	32.2 (6.4)	31.2 (7.2)	32.8 (5.8)
Picture Naming English (correct out of 30)	23.0 (3.6)	23.1 (3.3)	23.8 (3.2)
Picture Naming Spanish (correct out of 30)	16.3 (6.3)	13.0 (4.2)	13.6 (5.1)
Ratio Spanish/English naming correct	0.73 (0.32)	0.58 (0.22)	0.58 (0.24)
Autism Quotient (out of 50)	18.9 (7.3)	18.4 (6.1)	17.4 (5.0)

*Table lists means for each measure. Values in brackets are standard deviations unless noted otherwise. For explanation of the measures, see main text. ^∗^Differs from Exp. 1 group *p* < 0.05.*

#### Stimuli

One hundred and sixty pairs of sentences were constructed of the types illustrated in Table 2. *No Switch* conditions were in English only; *Switch* conditions started in English and switched to Spanish in the middle of the sentence. Since most of our participants were English dominant, the switch into the less-dominant language was expected to yield an LPC (see Litcofsky and Van Hell, 2017). We did not include sentences that started in Spanish. Having Spanish-only or Spanish-initial sentences would have meant introducing yet another type of confederate in Experiment 2, namely a Spanish monolingual peer. Adding such a confederate would be logistically difficult, and hard to make credible in a United States college context. In order to minimize potential differences in lexical frequency and semantics between the switch and no switch word, the critical position was always a highly frequent function word. Sentences were between nine and sixteen words in length, and the switch point varied between the 4th and 13th position. We did not have any filler items. In anticipation of Experiment 2, in which four conditions were used (Switch/No Switch crossed with presence of a monolingual or bilingual), sentences were Latin Squared over four lists, with 40 sentences for each of four virtual conditions. Note that we did not manipulate the presence of others in Experiment 1. We therefore collapsed over mono/bilingual present conditions in the analysis. In order to keep the participants engaged, 28% of the sentences were followed by a yes/no comprehension question about the preceding sentence. These questions were always presented in English only.

**TABLE 2:** Example of the materials.

Condition	Example
No Switch	The soccer player scored the winning goal in the last minute of the game.
Switch	The soccer player scored the winning goal en el último minuto del partido.
Y/N Question	Did the soccer team win by a landslide?

*Underscore indicates the critical position for which the ERP was analyzed. Y/N question is presented after the sentence.*

#### Procedure

The experiment consisted of two sessions. In the first session, participants completed a language background questionnaire, including questions on code-switching use. This information was used to describe participant characteristics, as well as confirm that the participants learned English and Spanish simultaneously, or Spanish first and English second. The participant was then given English and Spanish proficiency tasks, with the order of the language tested counterbalanced over participants. The English proficiency tasks were the grammar and cloze sections of the Michigan English Language Institute College Entrance Test (the MELICET), followed by a 30-item Boston Naming Task in English. The Spanish proficiency tasks were the Diplomas of Spanish as a Foreign Language (the DELE, a Spanish grammar task), followed by the Boston Naming Task in Spanish with 30 different pictures (Guzzardo Tamargo et al., 2016). Participants also completed a short form of the Edinburgh handedness inventory (Oldfield, 1971), and a short questionnaire to determine whether the participant has had epilepsy or other brain damage, or was currently taking medication that may affect the brain. In addition, participants filled out the Autism Spectrum Quotient (AQ, Baron-Cohen et al., 2001). The AQ is a questionnaire with 50 questions such as *I enjoy meeting new people*, yielding a score from 0 to 50 with a larger score indicating stronger autistic traits. The use of the measure was motivated by Jouravlev et al. (2019), who reported a trend for a smaller social N400 for those with stronger autistic traits as measured by this questionnaire.

The second session was on a separate day. In this session, participants were fitted with an electrode cap and read sentences while their EEG was recorded. Before the start of each sentence, a fixation cross appeared in the middle of the screen for 1000 ms. Sentences were presented one word at a time, in a white font on a black background, at a rate of 1 word every 500 ms (word presented for 300 ms followed by a 200 ms blank screen). Comprehension questions were presented after the last word and stayed on the screen until the participant answered yes or no by pushing the corresponding trigger buttons on a game pad. After each trial, the message “press for next” was presented. This stayed on the screen until the participant pressed a button on the gamepad. Sentences were presented in 8 blocks of 20 sentences with a short pause between the blocks. Before the actual task, a practice block was presented with five unilingual English sentences, three of which were followed by questions. After the study, participants completed a debriefing form with questions about their experience doing the task. The study took about 2.5 h in total per participant: 1 h for the first session, 1.5 for the second.

#### EEG Recording and Preprocessing

EEG was recorded from 64 Ag/AgCl electrodes mounted in an elastic cap (ANT-Neuro Waveguard^TM^). EEG was recorded at a sampling rate of 512 Hz, relative to an average reference using an ANT Refa 78 amplifier (ANT-Neuro, Hengelo, Netherlands). Eye movements were recorded from electrodes placed on the outer canthi, and above and below the right eye. Signal processing was done using EEGLab (Delorme and Makeig, 2004) and ERPLab (Lopez-Calderon and Luck, 2014) running on Matlab. The signal was referenced off-line to the mean of the left and right mastoids, and band-pass filtered between 0.01 and 30 Hz. In addition, trials with eye movements and other artifacts were automatically rejected (trials were rejected with VEOG amplitudes above 60 μV in a 200 ms window using 100 ms steps, with HEOG amplitudes above 45 μV in a 400 ms window using 50 ms steps, and with an overall amplitude smaller than −75 μV or larger than 75 μV). The average number of trials included in the analysis was 59.1 for the No-switch condition and 58.6 for the Switch condition. Epochs were 1200 ms long and spanned the interval from 200 ms before to 1000 ms after the onset of the code-switched word or its control. The 200 ms pre-stimulus window was used as baseline.

#### Analysis

For each artifact-free trial, we computed the average amplitude in the following time windows. First, the early frontal positivity was defined as the amplitude over 200–300 ms after word onset, averaged over ten electrodes: Fz/1/2/3/4 and FCz/1/2/3/4. The N400 was defined as the amplitude between 300 and 500 ms over Cz/1/2/3/4, FCz/1/2/3/4, CPz/1/2/3/4; the LAN as the amplitude between 300 and 500 ms over C1/3/5, FC1/3/5 and F1/3/5; and the LPC as 500–900 ms (Litcofsky and Van Hell, 2017) over Pz/1/2/3/4/5/6, CPz/1/2/3/4/5/6.

For each time window, we conducted a linear mixed-effects analysis using lme4 version 1.1-21 (Bates et al., 2015b) in R version 3.6.1 (R Core Team, 2019). Models were constructed with Switch, Half, and their interaction as fixed effects. Half was included as a fixed effect in order to test potential differences between the first and second half of the study. It was important to test the effects of the first versus the second half, since the two halves would be associated with different confederates in Experiment 2. Factors were deviation coded (No-switch −0.5, Switch +0.5; First half −0.5, Second half +0.5). We started with models that contained a full random effects structure. These models did not converge. We then took out Half from the random by-item slopes. We then tested the number of random effect parameters supported by rePCA (Bates et al., 2015a). Random effect structures were reduced by omitting factors with the smallest variance until the number of parameters was supported by the data and the model did not result in a singular fit. The final models for the LAN and N400 analyses had by-subject and by-item intercepts only; models for the early frontal positivity and LPC also had a by-subject random slope for Switch. *P*-values were estimated based on Satterthwaite’s method using LmerTest version 3.1-0 (Kuznetsova et al., 2017). Outcomes of all models are provided in the Supplementary Material.

### Results

#### Comprehension Questions

Participants completed the comprehension questions with high accuracy (Accuracy, Switch *M* = 0.91, *SD* = 0.07; No-switch *M* = 0.94, *SD* = 0.08), suggesting our participants were reading attentively. A logistic linear mixed-effects model with Switch as a fixed effect (deviation coded with No-switch as −0.5; Switch as 0.5), by-subject and by-item intercepts, and Switch as a by-item random slope yielded no effects of Switch (*b* = −1.23; *z* = −1.26, *p* > 0.2).

#### Event-Related Potentials

**FIGURE 1:**
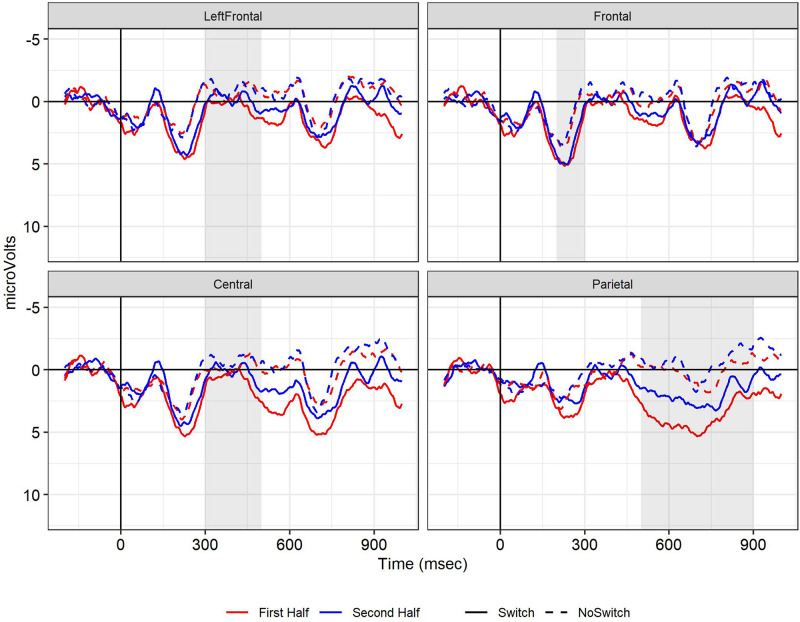
Experiment 1, grand mean waveforms for the Switch (solid line) and No-switch words (dotted line) over left frontal (LAN), central (N400), frontal (frontal positivity) and parietal (LPC) electrode sites used for analysis. Red line: first half, Blue line: second half. In this and other line graphs, negative is plotted up, the onset of the critical word is at 0 ms (*x*-axis), and shaded regions indicate the time windows used for statistical analysis.

Figure 1 displays the ERPs for the first and second half of the study for the combined electrodes to assess the LAN (left frontal), N400 (central), early frontal positivity (frontal) and LPC (parietal). Switch words elicited a larger positivity than no-switch controls starting at 200 ms.

##### Early frontal positivity (200–300 ms)

Switch words elicited a larger positivity between 200 and 300 ms at fronto-central sites compared to control words (*b* = 2.20, *SE* = 0.59, *t* = 3.75, *p* < 0.01). There were no effects of Half or an interaction between Switch and Half.

##### LAN and N400 (300–500 ms)

We did not observe a LAN or N400. Between 300 and 500 ms after onset of the critical word, ERPs became more *positive* for the Switch than No-switch conditions over a broad part of the scalp. The LAN analysis (300–500 ms, left frontal sites) and N400 analysis (300–500 ms, central sites) revealed an effect of Switch, but again, the effects were in the opposite direction of what was expected (LAN: *b* = 1.24, *SE* = 0.35, *t* = 3.51, *p* < 0.001; N400: *b* = 1.20, *SE* = 0.37, *t* = 3.26, *p* < 0.01). For the N400 window and region, ERPs in the second half were more negative than in the first half, regardless of Switch condition (*b* = −0.73, *SE* = 0.37, *t* = −1.98, *p* < 0.05).

##### LPC (500–900 ms)

In the 500–900 ms time window at parietal sites, Switch trials elicited a larger positivity than No-switch trials (*b* = 3.00, *SE* = 0.52, *t* = 5.76, *p* < 0.001). Overall, ERPs were less positive in the second half than the first (*b* = −1.54, *SE* = 0.36, *t* = −4.22, *p* < 0.001). Although the LPC Switch effect was numerically smaller in the second half (see Figure 1), there was no significant interaction between Half and Switch (*p* > 0.3).

### Discussion

Replicating other studies (Moreno et al., 2008; Van Der Meij et al., 2010; Ng et al., 2014; Litcofsky and Van Hell, 2017; Fernandez et al., 2019), we found an LPC for switch words versus control words. This positivity started already in the LAN/N400 time window. The lack of the N400 and LAN switch effects (that is, the lack of a negativity for the switch vs. no-switch conditions) could be due to the target words being function words rather than content words. However, Litcofsky and Van Hell (2017) did not report N400 or LAN for switches either, even though their target words were content words in visually presented sentences. Overall, the ERPs were less positive in the second half of the study, but this did not significantly affect the size of the switch effect. We also found an early positivity for the switch words versus control words. This replicates findings by Beatty-Martínez and Dussias (2017), who report an early positivity for switches for those participants who did not habitually code-switch themselves. Beatty-Martínez and Dussias (2017) interpret this component as a combination of a P2-N2 and P3a, reflecting a shift of attention from a narrow focus (one language) to a broader focus (both languages), cf. Green (2018). Although most of our participants were moderate code-switchers and indicated to be regularly exposed to code-switching, the use of switching on a function word in a written, isolated context in our study, may not have been expected or plausible enough to expect and pre-activate Spanish, or to have a broad attentional focus, necessitating an attentional shift.

## Experiment 2

The goal of Experiment 2 was to see to what extent the switch effects observed in Experiment 1 could be modulated by the co-presence of a Spanish-English bilingual or an English monolingual. The rationale is that code-switching is not socially allowed in the presence of a monolingual, which may affect the degree to which a switch is expected, or Spanish is co-activated. Since we found only the early frontal positivity and the LPC to be sensitive to code-switches in Experiment 1, we will focus on these two components in Experiment 2. We used the same materials as in Experiment 1, but had the participant read the sentences with a partner sitting beside them who they knew was either a monolingual English speaker or a bilingual Spanish-English speaker. These partners were trained confederates. Before the reading task, the participant was familiarized with the confederate partner and their language background by means of an interactive conversation task (map task, see details below).

### Materials and Methods

#### Participants

Thirty-nine healthy young adults, drawn from the same population as Experiment 1, participated in the study either for course credit or a $10/h monetary compensation. Data from six participants were omitted from the analysis because of artifacts (four participants had fewer than 20 artifact-free trials for one or more conditions), technical difficulties (one participant) or because they believed the monolingual confederate was a Spanish-English bilingual (one participant, see below). The remaining data set consisted of data from 33 participants. Sixteen of these participants started the ERP session with a monolingual confederate and switched to a bilingual confederate in the second half; seventeen had the reverse order. Participant characteristics are given in Table 1. The two groups in Experiment 2 did not differ from each other on any of the measures collected, as determined by *t*-tests. There were also no differences between the group in Experiment 1 and each of the groups in Experiment 2, except that the group who saw the bilingual partner first in Experiment 2 scored lower than the group in Experiment 1 on the extent they encountered code-switching in daily life.

As in Experiment 1, most of our participants were English dominant, except two participants who named more Spanish than English words in the naming tasks. Removing these two Spanish-dominant participants from analysis did not affect the results (see Supplementary Material). We therefore report results including these participants.

#### Stimuli and Procedure

Materials and EEG recording and preprocessing methods were the same as in Experiment 1. The procedure was similar, with the following changes. First, participants read the sentences with an English monolingual confederate sitting next to them in the booth for one half of the study and a Spanish-English bilingual in the other half. The confederates did not wear an electrode cap; EEG was recorded only from the participant. The order of confederates was reversed for about half of the participants. Over the course of the study, we had 6 monolingual confederates and 10 Spanish-English confederates, drawn from the same population as our participants. Confederates were all female, undergraduate or graduate students, aged 19–25. The monolingual English confederates all spoke American English with a standard accent; the Spanish-English confederates all had learned Spanish from birth and English before the age of 8 and reported to code-switch themselves in daily conversation. The participant was introduced to the confederate before each half by means of a map task, an interactive conversation task (Valdés Kroff and Fernández-Duque, 2017). In the map task, the confederate and participant each saw a stylized picture of a landscape with the same objects in different locations. Neither the confederate or participant had access to the other’s map. They took turns verbally instructing the other person where to place an object on a map aiming to come to the same configuration of objects in the end. This map task took about 5 min. Both confederates were instructed to engage in informal conversation with the participant such that language background would come up in conversation and they could mention that they either spoke no Spanish at all or were bilingual Spanish-English speakers themselves. Additionally, the bilingual confederate was instructed to occasionally code-switch to Spanish in the map task and during social conversation before the EEG session; the monolingual confederate was instructed to only speak English.

A second difference compared to Experiment 1 pertains to the reading study. After each sentence, participants and confederates were asked to indicate whether they thought the person next to them understood the sentence (cf. Rüschemeyer et al., 2015; Jouravlev et al., 2019). Both participant and confederate each held a game-pad to respond. We recorded responses from the participant only. In addition, the confederate was sitting slightly behind the participant, such that the participant could not observe the confederate’s responses. As in Experiment 1, 28% of the sentences was followed by a comprehension question. This question came after the meta-probe. After the participant answered the comprehension questions, they (and the confederate) were probed to indicate whether they thought the other had answered the question correctly. The participant and confederate were unaware of the other’s answers to any of the probes.

Third, after the study, the participant was debriefed. One of the debriefing questions was whether they thought the confederate did or did not speak Spanish and whether they thought the confederate was a naïve participant. Participants were then told about the confederates being set up by the experimenters and were asked to re-consent to the use of their data.

#### Analysis

Preprocessing procedures were the same as in Experiment 1 (average number of trials included in the analysis: monolingual partner, No-switch: 33.2; Switch 33.5; bilingual partner, No-switch: 32.8, Switch: 33.5). Since no LAN and N400 switch effects were obtained in Experiment 1, we will only report analyses of the early frontal positivity and the LPC. These components were quantified in the same manner as in Experiment 1. For each of the effects, a linear mixed-effects model was constructed with Switch, Half, Partner Type (Bilingual or Monolingual) and their interactions as fixed effects. Factors were deviation coded (No-switch −0.5, Switch +0.5; First half −0.5, Second half +0.5; Bilingual partner −0.5, Monolingual partner +0.5). We followed the procedure described in Experiment 1 to reduce the random-effects structure. Most models reported included by-subject and by-items random intercepts and Switch as a by-subject and by-item random slope. For the complete model description and outcomes, see the Supplementary Material.

### Results

#### Debriefing

Debriefing suggested that all participants believed that the monolingual confederate spoke no Spanish, whereas the bilingual confederate did (except one participant whose data were omitted from analysis). About half of the participants indicated in the debriefing that either the monolingual confederate (6 participants), the bilingual confederate (7), or both (5) may not have been naïve to the study. This impression was often based on the confederate appearing rather relaxed, on the observation that both confederates mentioned language in social conversation, or, in some cases, the impression that the bilingual confederate started code-switching out-of-the-blue. As the primary aim of the study concerns how knowledge of an interlocutor’s language background affects bilingual sentence processing, we did not omit participants on the basis of whether they thought the confederates were naïve to the study.

#### Behavioral Data

##### Comprehension questions

Participants performed slightly worse on the comprehension questions than in Experiment 1, possibly due to the dual-task and the fact that somebody was with them in the booth (Accuracy, Bilingual confederate, Switch: *M* = 0.89, *SD* = 0.09; No-switch: *M* = 0.89, *SD* = 0.06; Monolingual confederate, Switch: *M* = 0.86, *SD* = 0.11; No-switch: *M* = 0.89, *SD* = 0.10). We analyzed these and the other behavioral data reported below with a logistic mixed-effects model with Switch, Type of Partner, and their interaction as fixed effects, and by-subject and by-item random intercepts. Switch and Type of Partner were deviation coded (No-switch −0.5; Switch 0.5; Bilingual −0.5, Monolingual 0.5). This analysis yielded no significant effect of Switch, Type of Partner, or of an interaction between the two factors for the participants’ responses to comprehension questions (*p*s > *0.3*).

##### Did the partner answer the question correctly?

Participants were asked to indicate whether they thought the partner (confederate) had answered the question correctly. When the sentence was entirely in English, or when the partner was a bilingual and the sentence contained a switch, the response was overwhelmingly positive. When the partner was a monolingual English speaker and the sentence contained a switch to Spanish, participants responded “yes” in half of the cases on average (proportion of “yes” responses, Bilingual partner, Switch: *M* = 0.98, *SD* = 0.18; No-switch: *M* = 0.97, *SD* = 0.16; Monolingual partner, Switch: *M* = 0.51, *SD* = 0.50; No-switch: *M* = 0.98, *SD* = 0.15). This pattern yielded a significant interaction between Switch and Type of Partner (*b* = −4.17, *SE* = 0.63, *z* = −6.63, *p* < 0.001), as well as main effects of Switch (*b* = −2.32, *SE* = 0.31, *z* = −7.38, *p* < 0.001), and Type of Partner (*b* = −1.91, *SE* = 0.31, *z* = −6.22, *p* < 0.001).

##### Did the partner understand the sentence?

After each sentence, participants indicated whether they thought their partner understood the sentence. Participants indicated that their partner understood the sentence more often when the sentence was in English only than when it contained a switch. As expected, the proportion of “yes” responses was smallest for the switch condition with the monolingual partner (proportion of “yes” responses, Bilingual partner, Switch: *M* = 0.98, *SD* = 0.13; No-switch: *M* = 0.99, *SD* = 0.05; Monolingual partner, Switch: *M* = 0.41, *SD* = 0.49; No-switch: *M* = 0.99, *SD* = 0.07). The interaction between Switch and Type of Partner was significant (*b* = −5.02, *SE* = 0.75, *z* = −6.67, *p* < 0.001), as were the effects of Switch (*b* = −4.54, *SE* = 0.38, *z* = −12.08, *p* < 0.01) and Type of Partner (*b* = −3.32, *SE* = 0.37, *z* = −8.91, *p* < 0.01). We should note that there was a bimodal distribution in the partner-related responses in the switch condition with the monolingual partner. Nine of the 33 participants responded “yes” on more than 70% of the trials; 21 responded “yes” on 37.5% or fewer trials; the remaining three responded “yes” on 50 to 60% of the trials. This was not related to whether the monolingual confederate came first or second. Participants therefore either mostly did, or mostly did not take the partner’s language knowledge into consideration in their response^1^. Since sensitivity to the partner’s language knowledge is critical for what we want to show regarding language control, we will therefore report two analyses on the ERP data: one for all participants, and one including only those 21 that took their partner’s language knowledge into consideration most of the time when responding to the prompt.

#### Event-Related Potentials: All Participants

##### Early frontal positivity (200–300 ms)

**FIGURE 2:**
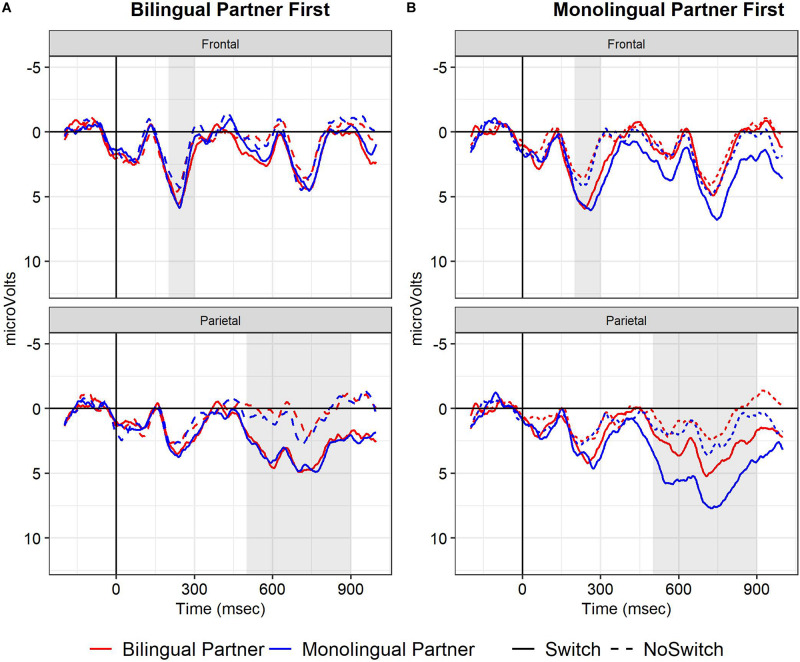
Experiment 2, ERPs at frontal and parietal sites (see main text) for participants that started the task with bilingual partner **(A)**, and those who started with a monolingual partner **(B)**. Grand mean waveforms for the Switch (solid line) and No-switch words (dotted line) when a monolingual (blue line), or bilingual partner (red line) was present.

**FIGURE 3:**
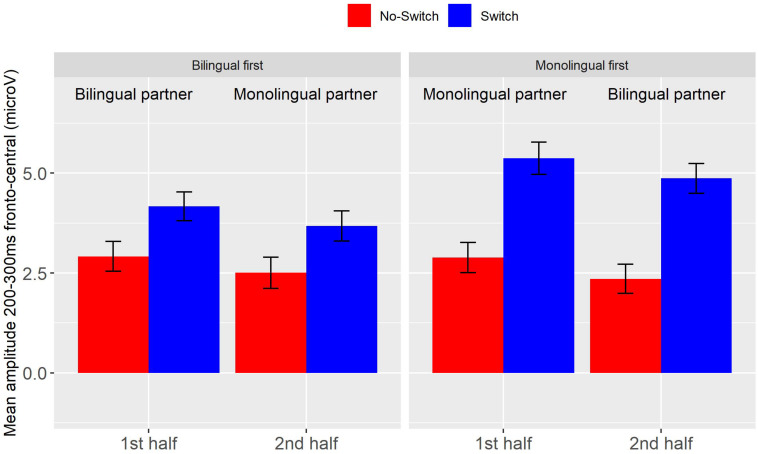
Experiment 2, average amplitude between 200 and 300 ms at fronto-central electrodes for the Switch (blue) and No-switch condition (red), per order group and half. Error bars are standard errors.

Event-related potentials for the mono- and bilingual partner conditions for all participants in the two order groups are depicted in Figure 2. As in Experiment 1, the early frontal positivity was larger for switch words than for control words (*b* = 1.84, *SE* = 0.33, *t* = 5.61, *p* < 0.001). In addition, the interaction of Switch by Half by Partner Type was significant (*b* = −2.71, *SE* = 1.24, *t* = −2.18, *p* < 0.05). This triple interaction was due to the switch effect being larger with a bilingual partner when present in the second half and larger with a monolingual partner when present in the first half of the study. Thus, participants who started the task with a monolingual participant showed a larger switch effect in both halves of the study. This can be seen in Figure 3 depicting the mean amplitude for the frontal region between 200 and 300 ms for the two order groups in each of the conditions and halves. The main purpose of our study was to investigate the effect of Partner Type on the Switch effect. Therefore, to better understand these effects within each of the order groups, we conducted follow-up analyses separately for both order groups, using Switch and Type of Partner, and their interactions as fixed effects. We found switch effects in both groups, with larger estimates in the monolingual-first group (Monolingual first: *b* = 2.47, *SE* = 0.37, *t* = 6.64, *p* < 0.001; Bilingual first: *b* = 1.22, *SE* = 0.37, *t* = 3.31, *p* < 0.001). Neither group showed effects involving Type of Partner; that is, we have no evidence that the switch effect or the positivity overall changed with a change of partner in the second half of the study, regardless of whether one started with a bilingual or a monolingual partner.

##### LPC (500–900 ms)

**FIGURE 4:**
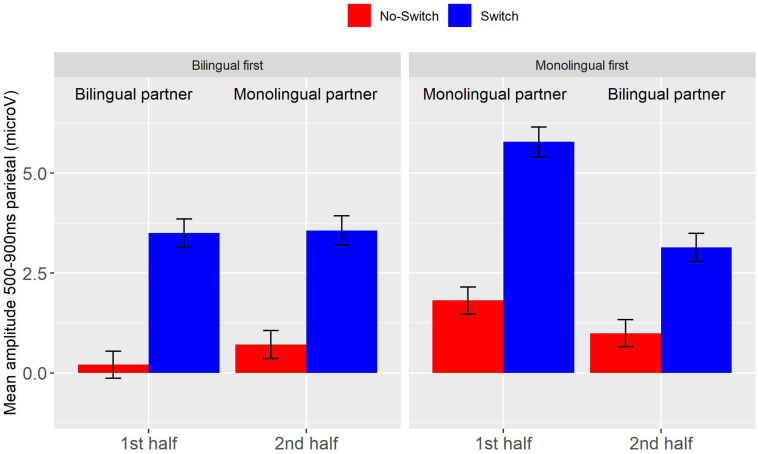
Experiment 2, average amplitude between 500 and 900 ms at centro-parietal electrodes for the Switch (blue) and No-switch condition (red), per order group and half. Error bars are standard errors.

As in Experiment 1, switch words elicited an LPC compared to no-switch control words (*b* = 3.04, *SE* = 0.44, *t* = 6.97, *p* < 0.001). In contrast to Experiment 1, the switch effect was significantly smaller in the second half than in the first (Switch by Half: *b* = −1.16, *SE* = 0.48, *t* = −2.41, *p* < 0.05). There were no significant interactions of Switch and Type of Partner. However, ERPs were overall more positive when a monolingual was present compared to a bilingual (*b* = 1.01, *SE* = 0.24, *t* = 4.19, *p* < 0.001), and were on average less positive in the second half than the first (*b* = −0.68, *SE* = 0.24, *t* = 2.81, *p* < 0.01). These effects are illustrated in Figure 4.

#### ERPs: Subset of Those Considering Partner’s Language Knowledge

**FIGURE 5:**
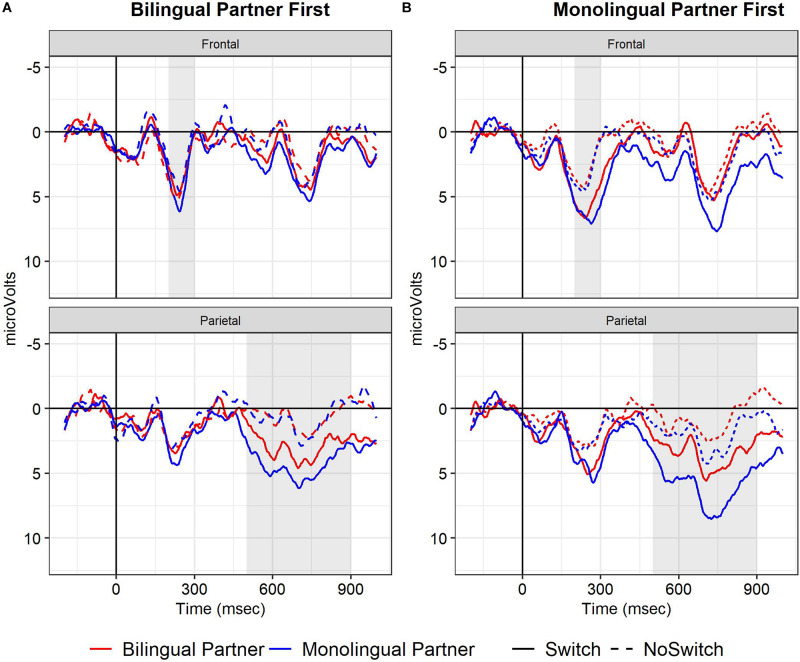
Experiment 2, ERPs of the subset of participants that were sensitive to their partner’s language knowledge. **(A)** Those who started the task with a bilingual partner (*n* = 9); **(B)** those who started with a monolingual partner (*n* = 12). Grand mean waveforms at frontal and parietal sites for the Switch (solid line) and No-switch words (dotted line) when a monolingual (blue line), or bilingual partner (red line) was present.

As mentioned in the discussion of the behavioral data, 21 participants responded 37.5% or less of the time that their monolingual partner understood the preceding sentence when it had a code-switch. To see to what extent this sensitivity to their partner’s language knowledge affected the ERPs, we conducted a second analysis in which we only included these 21 participants (nine had the bilingual confederate first, twelve the monolingual confederate)^2^. Figure 5 gives the ERPs for this subset of participants.

##### Early frontal positivity (200–300 ms)

**FIGURE 6:**
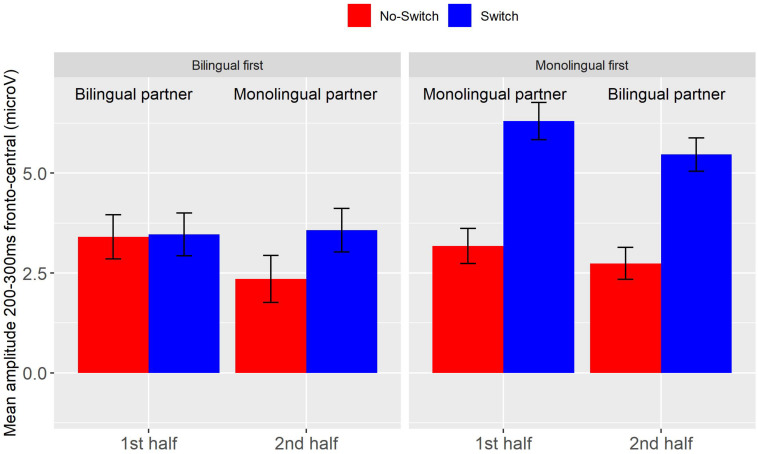
Experiment 2, data for the subset of participants that were sensitive to their partner’s language knowledge. Average amplitude between 200 and 300 ms at fronto-central electrodes for the Switch (blue) and No-switch condition (red), per order group and half. Error bars are standard errors.

The early frontal positivity switch effect was largest in those who did the task with a monolingual partner first. In contrast to the full data set, the early frontal positivity switch effect was absent in those who started the task with a bilingual partner. These effects are illustrated in Figure 6. A linear mixed-effects model with the same factors as in the analysis of the full data set again showed a main effect of Switch (*b* = 1.78, *SE* = 0.35 *t* = 5.03, *p* < 0.001), and a triple interaction of Switch by Type of Partner by Half (*b* = −4.55, *SE* = 1.35 *t* = −3.39, *p* < 0.001). Separate follow-up analyses for each of the order groups yielded a significant switch effect for those who first did the task with a monolingual partner (*b* = 2.89, *SE* = 0.43, *t* = 6.80, *p* < 0.001), but not for those who started with a bilingual partner (*b* = 0.65, *SE* = 0.54, *t* = 1.20, *p* > 0.2). Neither group showed a main effect of Type of Partner, similar to the main analysis.

##### LPC (500–900 ms)

**FIGURE 7:**
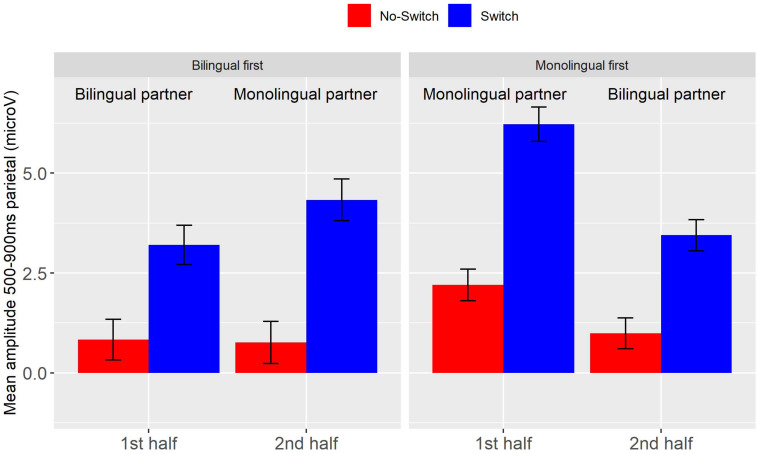
Experiment 2, data for the subset of participants that were sensitive to their partner’s language knowledge. Average amplitude between 500 and 900 ms at centro-parietal electrodes for the Switch (blue) and No-switch condition (red), per order group and half. Error bars are standard errors.

The LPC switch effect was smaller when the partner was a bilingual than a monolingual regardless of whether the monolingual partner was introduced in the first or the second half of the session (Switch by Type of Partner: *b* = 1.35, *SE* = 0.63, *t* = 2.14, *p* < 0.05). These effects are illustrated in Figure 7. The main effects found in the analysis of the full data set (Switch, Half, Type of Partner) remained significant.

### Discussion

Experiment 2 replicated the switch effects in Experiment 1: switch words elicited a larger early frontal positivity and LPC than non-switch controls. These effects were modulated by the presence of a bilingual or monolingual partner, but manifested in different ways. First, the LPC switch effect was smaller when a bilingual partner was present than when a monolingual partner was present, but only for those participants who indicated that they thought their monolingual partner did not understand the sentences with the switches. Second, the early frontal positivity switch effect was smaller for those participants who were first paired with a bilingual confederate. This effect did not change with a change of partner. The early positivity switch effect even disappeared for those participants who started with a bilingual partner and were sensitive to their partner’s language knowledge. However, given the small number of participants in the latter group (9) we are cautious in interpreting this effect. As in Experiment 1, our participants self-reported to code-switch on a regular basis. The early switch effect is therefore in apparent contrast with Beatty-Martínez and Dussias (2017) who report an early positivity for switches only for participants who do not code-switch themselves. As mentioned in the discussion of Experiment 1, this discrepancy can be due to the fact that our code-switches were rather unusual in that we switched from English to Spanish and switched on function words.

## General Discussion

### Summary

In the current study, we tested whether the co-presence of a bilingual or monolingual affected the processing of code-switches. In Experiment 1, which served as a control experiment, we found two switch effects: an early frontal positivity and an LPC. Experiment 2 tested whether bilinguals could adjust their expectation of the language used and of code-switching based on the language knowledge of a co-present person. If so, we expected the switch-effects in ERPs to be smaller in the presence of a bilingual than a monolingual. We found both the frontal positivity and the LPC switch effects to be sensitive to the co-presence of a bilingual versus monolingual, but in different ways. First, the early frontal positivity switch effect was smaller for those participants who first completed the task with a bilingual partner. Second, the LPC effect for switches vs. non-switches was smaller when a bilingual was present compared to a monolingual, but only when restricting the data analysis to participants who took their partner’s language knowledge into consideration when evaluating whether their partner understood the sentence. Below we will discuss these and relate these effects to models of bilingual language processing. We will conclude with some caveats and suggestions for further research.

### Early Frontal Positivity

Assuming that an early frontal positivity is associated with the efficiency of extracting visual features (Luck and Hillyard, 1994; Federmeier et al., 2005), shifts of attention from the expected to the unexpected language, or a shift from a narrow to a broad attentional focus (Beatty-Martínez and Dussias, 2017), the reduction or absence of an early frontal positivity switch effect in the presence of a bilingual versus monolingual suggests that bilinguals pro-actively adjusted the levels of activation of the languages or their attentional focus in the presence of another bilingual. When code-switched sentences were presented without a partner (Experiment 1) or with a monolingual English partner first (Experiment 2), the switch to Spanish may have been unexpected, resulting in an attentional shift (early frontal positivity switch effect).

Event-related potentials studies on language production (picture naming) have typically observed an early positivity as well. This positivity has been found to be sensitive to lexical factors such as word frequency and cognate status (Strijkers et al., 2009, 2011); however, the distribution of this positivity is more posterior than the positivity we report. We do not exclude that the frontal effects we observe reflect lexical factors. For instance, lexical access may have been less efficient in a switch than no-switch trial. Importantly, however, the switch effect was modulated by the presence of a bilingual or monolingual, while the lexical items themselves were kept the same across partner conditions. Hence, even if the early positivity is interpreted as reflecting lexical processes, our results suggest that this is modulated in a top-down and likely anticipatory fashion by the non-linguistic context.

The early frontal positivity effect in our study, however, did not change with a change of type of partner: the early frontal positivity switch effect remained larger in the second half when those in the monolingual-first group were partnered with a bilingual. The monolingual-first and bilingual-first groups were well-matched on code-switching habits and other aspects of the participants’ language background. It is therefore unlikely that this effect is due to group differences. Rather, the findings suggest that the processes reflected by the early frontal positivity are globally adjusted and do not change quickly when the situation allows a change. Note that such global adjustment is quite common in production studies. For instance, Christoffels et al. (2016) report that, after a language switching block, bilinguals continued to be slower naming items in the L1, even in a unilingual L1 context. This inhibition effect lasted for over 10 min. This suggests that L1 inhibition is sustained even when no longer needed in the context. Similarly, other research suggests that people tend to stick to their initial communication strategies, even when these are no longer required by the context (e.g., Vogels et al., 2019). Most of these studies involve production. Studies on word-level language switching in comprehension typically find no evidence for pro-active language control (Declerck, 2019; Declerck et al., 2019). Our results, however, suggest that bilinguals can pro-actively control language during comprehension in a sentence context, and, as in production, do not change their pro-active language control on a quick time scale. It, however, remains to be explained why bilinguals quickly accommodate their language activation to a bilingual partner at the start of a study, but not to a bilingual partner who is introduced halfway through the study. One factor may be loss of sensitivity to the non-linguistic context over the course of the study, perhaps due to fatigue. Future studies, in which the two partner sessions are separated by a few days or weeks, could shed more light on the time scale of this pro-active control in comprehension.

### LPC Effects

The LPC has been interpreted as reflecting later stage processes such as sentence-level revision (Litcofsky and Van Hell, 2017) or stimulus evaluation and memory updating in response to unexpected events (Moreno et al., 2008). We found an LPC for code-switched words in both Experiment 1 and 2. This suggests that the LPC switch effect cannot be fully attributed to the meta-cognitive task used in Experiment 2. Crucial for our main research question, the LPC was modulated by the type of partner present: for those participants who took the partner’s language knowledge into consideration, the LPC switch effects were smaller when a bilingual was present than when a monolingual was present regardless of the order in which the partners were introduced.

Our results suggest that the late revision or updating processes as reflected by the LPC can be dynamically adapted to the specifics of the context. If the use of a code-switch is socially more unexpected since the partner is a monolingual, the LPC is larger than when the switch is socially appropriate. This ties into findings that the LPC is sensitive to social norms and is larger for stimuli that are socially unexpected or negative (Lattner and Friederici, 2003; Schupp et al., 2003, 2004; Schacht and Sommer, 2009; Fields and Kuperberg, 2012). The fact that the LPC switch effect is related to the participants’ responses as to their partner’s understanding suggests that the adaptation of the updating processes to the non-linguistic context is not automatic. This is not surprising, since many late processes reflected by ERPs are typically modulated by tasks and strategies. For instance, the P600 found for syntactic violations is modulated by the number of ungrammatical distractor items in the stimulus set (Coulson et al., 1998; Hahne and Friederici, 1999), and whether the participants are asked to make a grammaticality judgment or read the sentence for comprehension (e.g., Kaan and Swaab, 2003).

An additional account of the LPC is that it reflects global language activation. In bilingual production studies using single words, a dominant language is typically responded to more slowly in mixed than in unilingual language contexts (Christoffels et al., 2007, 2016). Correspondingly, in production studies using EEG, the LPC has been reported to be smaller (less positive) for the dominant language in a mixed than a unilingual context (Timmer et al., 2019). This has been attributed to the overall inhibition of the dominant language in the mixed-language contexts in production^3^. In our study, the target word was always in (non-dominant) Spanish in switch trials, and (dominant) English in the no-switch trials. We can therefore not exclude that the LPC is at least in part driven by differences in global language activation. Our observation that LPC is less positive overall in the second half of both Experiment 1 and 2 supports this interpretation. After seeing many trials containing switches to Spanish, our participants may have been more likely to globally inhibit English and/or activate Spanish. However, even if the LPC switch effect can be ascribed to differences in global language activation, our results suggest that this language control is affected by the co-presence of a bi- or monolingual.

One potential concern is that what appears to be partner-specific effects in our study are actually due to the exposure to Spanish, or the absence of Spanish, in the map task prior to the EEG session. Since the bilingual confederates on occasion used some Spanish words in the map task, Spanish may have been primed when interacting with the bilingual partner. Spanish may therefore have been more active from the onset in this half of the EEG study, leading to smaller switch effects. However, the effects we observed cannot be completely due to priming of Spanish in the bilingual partner conditions. By the start of the second half of the study, all participants have been exposed to a great deal of Spanish words in the experimental materials. Priming by Spanish words can therefore not explain the larger LPC effects in monolingual partner condition in those who did the task with a bilingual partner in the first half and with a monolingual in the second half. Furthermore, if priming were the sole factor, one would also expect the early frontal positivity switch effect to be smaller for the bilingual versus the monolingual partner condition regardless of the order in which the confederates were introduced, since Spanish is used in the map task just before each EEG session with a bilingual partner. Our results are therefore at least in part driven by the knowledge that the partner was a bilingual or a monolingual, rather than solely by recent exposure to Spanish.

Our participants differed in the extent to which they considered their partner’s language background in responding whether their partner understood the sentence. These differences in behavior did not relate to the participants’ scores on the AQ (see text footnote 1). This is in contrast to Jouravlev et al. (2019), who reported a trend toward a smaller social N400 effects for those who had stronger autistic tendencies according to the questionnaire. This suggests that different mechanisms are involved in considering a person’s knowledge of language (our study) versus considering a person’s knowledge of a semantic context (Jouravlev et al., 2019) and that the AQ does not tap into the former. Future research should include additional varied measures of socio-cognitive skills and traits to see what underlies people’s ability or willingness to consider others’ language knowledge.

In sum, our results support the view that language control has different components that are differently modulated by the co-presence of a bilingual or monolingual. Pro-active control is related to the expectation of the use of both languages in context. In the current study, this is specifically related to the expectation of Spanish being used. With a bilingual partner at the start of the study, this expectation was apparently stronger, which meant that no large attentional shift (early frontal positivity) was needed when a Spanish word was encountered. This pro-active control was global, that is, did not change with a change of partner. The LPC can be associated with the degree to which a switch is expected and may reflect sentence revision and/or updating of this expectation. Assuming that sentence revision and context updating are easier when code-switching is socially permitted and is already more expected, revision and updating in response to switches were easier when a bilingual was present than when a monolingual was present. This led to a smaller LPC switch effect in the former situation.

### Models of Bilingual Comprehension

Models of bilingual processing can be extended to account for our findings in the following way. According to the BIA+ model (Dijkstra and Van Heuven, 2002) and Multilink model (Dijkstra et al., 2019b) of bilingual comprehension, non-linguistic factors such as the identity of a conversation partner can be assumed to affect the task/decision system, which cannot affect the activation in the lexicon. It will be hard in such a model to account for the pro-active effects that we have observed. To account for our observations, these models would need to incorporate a top-down effect from the task/decision system onto language activation within the lexicon such that it can pro-actively increase or lower the global activation threshold of items of a particular language. This can be done through e.g., a language node as in the BIA model that preceded the BIA+ (Dijkstra and Van Heuven, 1998). Data from other research supports such top-down activation as well (e.g., Hoversten et al., 2015; Martin et al., 2016). In addition to this top-down activation, the post-lexical decision processes in response to the code-switches need to be adapted depending on the context and partner. This can then account for the smaller switch effects in the later ERP components when the partner is bilingual compared to a monolingual.

The CPM (Green and Wei, 2014; Green, 2018) incorporates the idea that language control can be pro-actively adapted on the basis of the linguistic and non-linguistic context. This model distinguishes between competitive and cooperative control. Competitive control is implied in dual language situations in which only one language is used. It involves a narrow attentional state, in which one language is in the focus of attention. Cooperative control is implied in code-switching and is associated with a broad attentional state. The early frontal positivity could then be reflective of the attentional state that is pro-actively induced by the social context and, in the case of comprehension, modulates the activation of the lexical representation. However, one needs to assume that these attentional modes are not easily changeable, since the early frontal effects did not change with a change of partner. At the same time, the LPC was responsive to rapid changes in the context. Our results are therefore compatible with a CPM in which different language control processes are associated with different control mechanisms that can operate simultaneously on various scales.

### Caveats and Conclusion

We acknowledge that our task is not representative of natural language conversations in many ways, and we are therefore careful in generalizing the current results. For one, the code-switches used are rather uncommon. Spanish-English bilinguals with similar demographic characteristics to our sample tend to switch more from Spanish to English rather than vice-versa and tend to switch more in spoken than written language (Valdés Kroff et al., 2018). Furthermore, most switches occur on content words rather than function words (Poplack, 1980). The early frontal positivity switch effect in particular may have been due to the switch, or type of switch, being rather uncommon. Second, the modulation of the switch effects by the type of partner present may have been driven by the meta-cognitive task (*Did your partner understand?*). Jouravlev et al. (2019) report that the N400 is modulated by the co-presence of others only if the participants were asked whether the sentences made sense (in general or to the partner). Further research using more naturalistic social modulations (e.g., an interactive conversation task with various partners) is obviously needed. Furthermore, this line of research could be expanded to include more individual differences measures (with respect to e.g., Theory of Mind). Also, the perceived proficiency of the partner could be manipulated (Kapiley and Mishra, 2018) as well as the partner’s perceived code-switching habits. This could be done by extending the introductory interactive task (map-task) between the participant and confederate. Nevertheless, our finding that both the early frontal positivity and later switch effects (LPC) are modulated by the language knowledge of a co-present partner, but not in the same way, suggests that language control in comprehension involves various components that are differently recruited to accommodate to the non-linguistic context (Morales et al., 2013). This supports a dynamic control model of bilingual language comprehension.

## Data Availability Statement

The datasets generated for this study can be found on the Open Science Framework, https://osf.io/9hnxc/.

## Ethics Statement

This study was carried out in accordance with the recommendations of the University of Florida Institutional Review Board guidelines. The protocol was approved by the University of Florida Institutional Review Board. All subjects gave written informed consent in accordance with the Declaration of Helsinki.

## Author Contributions

EK and JV contributed to the conception and design of the study. EK, SK, AK, and AT were involved in implementing the study, data collection, and data preprocessing. EK and AT performed the statistical analysis. EK wrote the first draft of the manuscript. All authors contributed to manuscript revision, read, and approved the submitted version.

## Conflict of Interest

The authors declare that the research was conducted in the absence of any commercial or financial relationships that could be construed as a potential conflict of interest.

## Footnotes

1 There was no correlation between a participant’s score on the Autism Spectrum Quotient (AQ) and their responses with respect to the monolingual partner’s understanding of a code-switched sentence. Similarly, the LPC switch effect in the monolingual partner condition did not correlate with AQ scores. See Supplementary Material.

2 A potential objection is that this sample is not balanced in terms of confederate order. We therefore also analyzed data from the 24 participants who responded “yes” to *Did your partner understand?* on 60% or fewer of the code-switch trials in the monolingual partner condition. This sample consisted of 12 participants per order group. Results were the same as those reported in the main text, see Supplementary Material.

3 On the other hand, an eye-tracking study on sentence comprehension found overall faster reading time measures for both code-switched and unilingual sentences in mixed versus blocked contexts (Johns et al., 2019), suggesting that global language activation works differently in sentence comprehension than in single word production.
