# Supplementary material for: Processing Code-Switches in the Presence of Others: An ERP Study
Source: Front Psychol. 2020 Jun 26;11:1288. doi: 10.3389/fpsyg.2020.01288 (PMC7333233; doi:10.3389/fpsyg.2020.01288)
Supplement: Supplementary file 1 [file Data_Sheet_1.DOCX]

Supplementary Material

# Behavioral data: Outcomes of statistical models reported in the main text

## Experiment 1, Question accuracy

| ***Table S.1. Accuracy of comprehension questions, Logistic Linear mixed-effects model, Experiment 1*** | | | | |
| --- | --- | --- | --- | --- |
|  | **Accuracy** | | | |
| *Parameters* | *Estimate* | *SE* | *z* | *p* |
| Intercept | 3.99 ^***^ | 0.60 | 6.68 | **<0.001** |
| Switch | -1.23 | 0.98 | -1.26 | 0.207 |
| N _Subject_ | 16 | | | |
| N *_ItemNr_* | 40 | | | |
| Observations | 640 | | | |
| Marginal R^2^ / Conditional R^2^ | 0.049 / 0.578 | | | |
| Accuracy ~ Switch + (1 \| Subject) + (1 + Switch \| ItemNr); ** p<0.05   ** p<0.01   *** p<0.001* | | | | |

## Experiment 2, Question accuracy

| ***Table S.2. Accuracy of comprehension questions, Logistic Linear mixed-effects model, Experiment 2*** | | | | |
| --- | --- | --- | --- | --- |
|  | **Accuracy** | | | |
| *Parameters* | *Estimate* | *SE* | *z* | *p* |
| Intercept | 2.34 ^***^ | 0.19 | 12.07 | **<0.001** |
| Switch | -0.13 | 0.17 | -0.74 | 0.457 |
| Partner | -0.16 | 0.18 | -0.89 | 0.372 |
| Switch:Partner | -0.13 | 0.35 | -0.37 | 0.710 |
| N _Subject_ | 33 | | | |
| N *_ItemNr_* | 40 | | | |
| Observations | 1316 | | | |
| Marginal R^2^ / Conditional R^2^ | 0.003 / 0.228 | | | |
| Accuracy ~ Switch * Partner + (1 \| Subject) + (1 *\| ItemNr*); ** p<0.05   ** p<0.01   *** p<0.001* | | | | |

## Experiment 2, ‘yes’ to *Did your partner answer the question correctly?*

| ***Table S.3. Did your partner answer the question correctly?, Logistic Linear mixed-effects model, Experiment 2*** | | | | |
| --- | --- | --- | --- | --- |
|  | **Yes-answers** | | | |
| *Parameters* | *Estimate* | *SE* | *z* | *p* |
| Intercept | 3.42 ^***^ | 0.37 | 9.29 | **<0.001** |
| Switch | -2.32 ^***^ | 0.31 | -7.38 | **<0.001** |
| Partner | -1.94 ^***^ | 0.31 | -6.22 | **<0.001** |
| Switch:Partner | -4.17 ^***^ | 0.63 | -6.63 | **<0.001** |
| N _Subject_ | 33 | | | |
| N *_ItemNr_* | 40 | | | |
| Observations | 1316 | | | |
| Marginal R^2^ / Conditional R^2^ | 0.353 / 0.657 | | | |
| QpartnerpromptYES ~ Switch * Partner + (1 \| Subject) + (1 \| ItemNr); ** p<0.05   ** p<0.01   *** p<0.001* | | | | |

##

## Experiment 2, ‘yes’ to *Did your partner understand the sentence?*

| ***Table S.4. Did your partner understand the sentence?, Logistic Linear mixed-effects model, Experiment 2*** | | | | |
| --- | --- | --- | --- | --- |
|  | **Yes-answers** | | | |
| *Parameters* | *Estimate* | *SE* | *z* | *p* |
| Intercept | 4.94 ^***^ | 0.48 | 10.29 | **<0.001** |
| Switch | -4.54 ^***^ | 0.38 | -12.08 | **<0.001** |
| Partner | -3.32 ^***^ | 0.37 | -8.91 | **<0.001** |
| Switch:Partner | -5.02 ^***^ | 0.75 | -6.67 | **<0.001** |
| N _Subject_ | 33 | | | |
| N *_ItemNr_* | 160 | | | |
| Observations | 5260 | | | |
| Marginal R^2^ / Conditional R^2^ | 0.502 / 0.826 | | | |
| PromptRePartnerYES ~ Switch * Partner + (1 \| Subject) + (1 \| ItemNr); ** p<0.05   ** p<0.01   *** p<0.001* | | | | |

# EEG data: Outcomes of statistical models reported in the main text

## Experiment 1, Early Frontal Positivity

| ***Table S.5 Linear mixed-effects model, Experiment 1, n=16*** | | | | |
| --- | --- | --- | --- | --- |
|  | **Amplitude 200-300 ms, Fronto-central sites** | | | |
| *Parameters* | *Estimate* | *SE* | *t* | *p* |
| (Intercept) | 2.52 ^***^ | 0.46 | 5.43 | **<0.001** |
| Switch | 2.20 ^***^ | 0.59 | 3.75 | **<0.001** |
| Half | -0.56 | 0.40 | -1.41 | 0.158 |
| Switch:Half | 0.13 | 0.79 | 0.16 | 0.872 |
| N _Subject_ | 16 | | | |
| N _ItemNr_ | 160 | | | |
| Observations | 1879 | | | |
| Marginal R^2^ / Conditional R^2^ | 0.016 / 0.068 | | | |
| Amplitude ~ Switch * Half + (1 + Switch \| Subject) + (1 \| ItemNr); ** p<0.05   ** p<0.01   *** p<0.001* | | | | |

## Experiment 1, LAN

| ***Table S.6 Linear mixed-effects model, Experiment 1, n=16*** | | | | |
| --- | --- | --- | --- | --- |
|  | **Amplitude 300-500 ms, Left Frontal sites** | | | |
| *Parameters* | *Estimate* | *SE* | *t* | *p* |
| Intercept | -0.43 | 0.25 | -1.71 | 0.106 |
| Switch | 1.24 ^***^ | 0.35 | 3.51 | **<0.001** |
| Half | -0.56 | 0.35 | -1.58 | 0.115 |
| Switch:Half | 0.43 | 0.71 | 0.61 | 0.544 |
| N _Subject_ | 16 | | | |
| N _ItemNr_ | 160 | | | |
| Observations | 1884 | | | |
| Marginal R^2^ / Conditional R^2^ | 0.008 / 0.024 | | | |
| Amplitude ~ Switch * Half + (1 \| Subject) + (1 \| ItemNr); ** p<0.05   ** p<0.01   *** p<0.001* | | | | |

## Experiment 1, N400

| ***Table S.7 Linear mixed-effects model, Experiment 1, n=16*** | | | | |
| --- | --- | --- | --- | --- |
|  | **Amplitude 300-500 ms, Central sites** | | | |
| *Parameters* | *Estimate* | *SE* | *t* | *p* |
| Intercept | 0.01 | 0.28 | 0.04 | 0.966 |
| Switch | 1.20 ^**^ | 0.37 | 3.26 | **0.001** |
| Half | -0.73 ^*^ | 0.37 | -1.98 | **0.048** |
| Switch:Half | 0.50 | 0.74 | 0.68 | 0.495 |
| N _Subject_ | 16 | | | |
| N _ItemNr_ | 160 | | | |
| Observations | 1884 | | | |
| Marginal R^2^ / Conditional R^2^ | 0.008 / 0.024 | | | |
| Amplitude ~ Switch * Half + (1 \| Subject) + (1 \| ItemNr); ** p<0.05   ** p<0.01   *** p<0.001* | | | | |

## Experiment 1, LPC

| ***Table S.8 Linear mixed-effects model, Experiment 1, n=16*** | | | | |
| --- | --- | --- | --- | --- |
|  | **Amplitude 500-900 ms, Central-Parietal sites** | | | |
| *Parameters* | *Estimate* | *SE* | *t* | *p* |
| Intercept | 1.41 ^***^ | 0.36 | 3.92 | **<0.001** |
| Switch | 3.00 ^***^ | 0.52 | 5.76 | **<0.001** |
| Half | -1.54 ^***^ | 0.36 | -4.22 | **<0.001** |
| Switch:Half | -0.64 | 0.73 | -0.88 | 0.381 |
| N _Subject_ | 16 | | | |
| N _ItemNr_ | 160 | | | |
| Observations | 1879 | | | |
| Marginal R^2^ / Conditional R^2^ | 0.043 / 0.090 | | | |
| Ampl ~ Switch * Half + (1 + Switch \| Subject) + (1 \| ItemNr);** p<0.05   ** p<0.01   *** p<0.001* | | | | |

## Experiment 2, Early Frontal Positivity

### All, n=33

| ***Table S.9 Linear mixed-effects model, Experiment 2, n=33*** | | | | |
| --- | --- | --- | --- | --- |
|  | **Amplitude 200-300 ms, Fronto-central sites** | | | |
| *Parameters* | *Estimate* | *SE* | *t* | *p* |
| Intercept | 3.63 *** | 0.35 | 10.34 | **<0.001** |
| Switch | 1.84 *** | 0.33 | 5.61 | **<0.001** |
| Partner | 0.10 | 0.26 | 0.38 | 0.704 |
| Half | -0.46 | 0.26 | -1.78 | 0.075 |
| Switch:Partner | -0.08 | 0.52 | -0.15 | 0.878 |
| Switch:Half | 0.02 | 0.52 | 0.04 | 0.97 |
| Partner:Half | -1.2 | 1.36 | -0.88 | 0.378 |
| Switch:Partner:Half | -2.71 * | 1.24 | -2.18 | **0.029** |
| N _Subject_ | 33 | | | |
| N _ItemNr_ | 160 | | | |
| Observations | 4392 | | | |
| Marginal R^2^ / Conditional R^2^ | 0.014 / 0.080 | | | |
| Amplitude ~ Switch * Partner * Half + (1 + Switch \| Subject) + (1 + Switch \| ItemNr); ** p<0.05   ** p<0.01   *** p<0.001* | | | | |

### Bilingual first group, n=17

| ***Table S.10. Linear mixed-effects model, Experiment 2, bilingual first*** | | | | |
| --- | --- | --- | --- | --- |
|  | **Amplitude 200-300 ms, Fronto-central sites** | | | |
| *Parameters* | *Estimate* | *SE* | *t* | *p* |
| (Intercept) | 3.35 ^***^ | 0.48 | 6.94 | **<0.001** |
| Switch | 1.22 ^***^ | 0.37 | 3.31 | **0.001** |
| Partner | -0.35 | 0.37 | -0.95 | 0.342 |
| Switch: Partner | -0.13 | 0.74 | -0.18 | 0.858 |
| N _Subject_ | 17 | | | |
| Observations | 2212 | | | |
| Marginal R^2^ / Conditional R^2^ | 0.005 / 0.048 | | | |
| Amplitude ~ Switch * Partner + (1 \| Subject);** p<0.05   ** p<0.01   *** p<0.001* | | | | |

### Monolingual first group, n=16

| ***Table S.11. Linear mixed-effects model, Experiment 2, monolingual first*** | | | | |
| --- | --- | --- | --- | --- |
|  | **Amplitude 200-300 ms, Fronto-central sites** | | | |
| *Parameters* | *Estimate* | *SE* | *t* | *p* |
| (Intercept) | 3.94 ^***^ | 0.49 | 8.02 | **<0.001** |
| Switch | 2.47 ^***^ | 0.37 | 6.64 | **<0.001** |
| Partner | 0.51 | 0.37 | 1.36 | 0.174 |
| Switch: Partner | -0.06 | 0.75 | -0.08 | 0.934 |
| N _Subject_ | 16 | | | |
| N _ItemNr_ | 160 | | | |
| Observations | 2180 | | | |
| Marginal R^2^ / Conditional R^2^ | 0.020 / 0.085 | | | |
| Amplitude ~ Switch * Partner + (1 \| Subject) + (1 \| ItemNr); ** p<0.05   ** p<0.01   *** p<0.001* | | | | |

## Experiment 2, LPC, n=33

| ***Table S.12 Linear mixed-effects model, Experiment 2, n=33*** | | | | |
| --- | --- | --- | --- | --- |
|  | **Amplitude 500-900 ms, Central-Parietal sites** | | | |
| *Parameters* | *Estimate* | *SE* | *t* | *p* |
| Intercept | 2.48 ^***^ | 0.33 | 7.58 | **<0.001** |
| Switch | 3.04 ^***^ | 0.44 | 6.97 | **<0.001** |
| Partner | 1.01 ^***^ | 0.24 | 4.19 | **<0.001** |
| Half | -0.68 ^**^ | 0.24 | -2.81 | **0.005** |
| Switch:Partner | 0.72 | 0.48 | 1.5 | 0.135 |
| Switch:Half | -1.16 ^*^ | 0.48 | -2.41 | **0.016** |
| Partner:Half | -1.98 | 1.27 | -1.56 | 0.118 |
| Switch:Partner:Half | -0.19 | 1.68 | -0.11 | 0.912 |
| N _Subject_ | 33 | | | |
| N _ItemNr_ | 160 | | | |
| Observations | 4392 | | | |
| Marginal R^2^ / Conditional R^2^ | 0.043 / 0.119 | | | |
| Ampl ~ Switch * Partner * Half + (1 + Switch \| Subject) + (1 + Switch \| ItemNr); ** p<0.05   ** p<0.01   *** p<0.001* | | | | |

# Outcomes of statistical models for those with <37.5% “yes’ responses to “Did your partner understand the sentence?” in the code-switch condition with monolingual partner (Exp 2 only)

## Experiment 2, Early Frontal Positivity, n=21

| ***Table S.13 Linear mixed-effects model, Experiment 2, n=21*** | | | | |
| --- | --- | --- | --- | --- |
|  | **Amplitude 200-300 ms, Fronto-central sites** | | | |
| *Parameters* | *Estimate* | *SE* | *t* | *p* |
| Intercept | 3.88 ^***^ | 0.47 | 8.22 | **<0.001** |
| Switch | 1.78 ^***^ | 0.35 | 5.03 | **<0.001** |
| Partner | 0.17 | 0.34 | 0.49 | 0.626 |
| Half | -0.46 | 0.34 | -1.37 | 0.172 |
| Switch:Partner | 0.64 | 0.68 | 0.95 | 0.343 |
| Switch:Half | 0.44 | 0.67 | 0.65 | 0.518 |
| Partner:Half | -2.57 | 1.85 | -1.39 | 0.164 |
| Switch:Partner:Half | -4.55 ^***^ | 1.35 | -3.38 | **0.001** |
| N _Subject_ | 21 | | | |
| N _ItemNr_ | 160 | | | |
| Observations | 2794 | | | |
| Marginal R^2^ / Conditional R^2^ | 0.022 / 0.090 | | | |
| Amplitude ~ Switch * Partner * Half + (1 \| Subject) + (1 + Switch \| ItemNr); ** p<0.05   ** p<0.01   *** p<0.001* | | | | |

### Bilingual first group, n=9

| ***Table S.14. Linear mixed-effects model, Experiment 2, bilingual first*** | | | | |
| --- | --- | --- | --- | --- |
|  | **Amplitude 200-300 ms, Fronto-central sites** | | | |
| *Parameters* | *Estimate* | *SE* | *t* | *p* |
| (Intercept) | 3.24 ^***^ | 0.84 | 3.84 | **<0.001** |
| Switch | 0.65 | 0.54 | 1.20 | 0.229 |
| Partner | -0.29 | 0.54 | -0.53 | 0.593 |
| Switch: Partner | 1.04 | 1.08 | 0.96 | 0.336 |
| N _Subject_ | 9 | | | |
| Observations | 1142 | | | |
| Marginal R^2^ / Conditional R^2^ | 0.002 / 0.067 | | | |
| Amplitude ~ Switch * Partner + (1 \| Subject); ** p<0.05   ** p<0.01   *** p<0.001* | | | | |

### Monolingual first group, n=12

| ***Table S.15. Linear mixed-effects model, Experiment 2, monolingual first*** | | | | |
| --- | --- | --- | --- | --- |
|  | **Amplitude 200-300 ms, Fronto-central sites** | | | |
| *Parameters* | *Estimate* | *SE* | *t* | *p* |
| (Intercept) | 4.51 ^***^ | 0.51 | 8.87 | **<0.001** |
| Switch | 2.89 ^***^ | 0.43 | 6.80 | **<0.001** |
| Partner | 0.62 | 0.43 | 1.45 | 0.147 |
| Switch: Partner | 0.24 | 0.85 | 0.28 | 0.782 |
| N _Subject_ | 12 | | | |
| N _ItemNr_ | 160 | | | |
| Observations | 1652 | | | |
| Marginal R^2^ / Conditional R^2^ | 0.028 / 0.083 | | | |
| Amplitude ~ Switch * Conf + (1 \| Subject) + (1 \| ItemNr);** p<0.05   ** p<0.01   *** p<0.001* | | | | |

## Experiment 2, LPC, n=21

| ***Table S.16 Linear mixed-effects model, Experiment 2, n=21*** | | | | |
| --- | --- | --- | --- | --- |
|  | **Amplitude 500-900 ms, Central-Parietal sites** | | | |
| *Parameters* | *Estimate* | *SE* | *t* | *p* |
| Intercept | 2.74 ^***^ | 0.37 | 7.36 | **<0.001** |
| Switch | 3.08 ^***^ | 0.53 | 5.79 | **<0.001** |
| Partner | 1.29 ^***^ | 0.32 | 4.07 | **<0.001** |
| Half | -0.66 ^*^ | 0.31 | -2.09 | **0.036** |
| Switch:Partner | 1.35 ^*^ | 0.63 | 2.14 | **0.032** |
| Switch:Half | -0.18 | 0.63 | -0.29 | 0.774 |
| Partner:Half | -1.94 | 1.46 | -1.32 | 0.186 |
| Switch:Partner:Half | -0.64 | 2.09 | -0.31 | 0.76 |
| N _Subject_ | 21 | | | |
| N _ItemNr_ | 160 | | | |
| Observations | 2794 | | | |
| Marginal R^2^ / Conditional R^2^ | 0.045 / 0.103 | | | |
| Amplitude ~ Switch * Partner * Half + (1 + Switch \| Subject) + (1 + Switch \| ItemNr); ** p<0.05   ** p<0.01   *** p<0.001* | | | | |

# Outcomes of statistical models for those with <60% “yes’ responses to “Did your partner understand the sentence?” in the code-switch condition with monolingual Partner (Exp 2 only)

## Experiment 2, Early Frontal Positivity, n=24

| ***Table S.17 Linear mixed-effects model, Experiment 2, n=24*** | | | | |
| --- | --- | --- | --- | --- |
|  | **Amplitude 200-300 ms, Fronto-central sites** | | | |
| *Parameters* | *Estimate* | *SE* | *t* | *p* |
| Intercept | 3.98 ^***^ | 0.42 | 9.45 | **<0.001** |
| Switch | 2.09 ^***^ | 0.33 | 6.37 | **<0.001** |
| Partner | 0.06 | 0.31 | 0.2 | 0.844 |
| Half | -0.56 | 0.31 | -1.79 | 0.073 |
| Switch:Partner | 0.38 | 0.63 | 0.6 | 0.547 |
| Switch:Half | 0.13 | 0.62 | 0.2 | 0.84 |
| Partner:Half | -2.18 | 1.65 | -1.32 | 0.188 |
| Switch:Partner:Half | -3.42 ^**^ | 1.25 | -2.74 | **0.006** |
| N _Subject_ | 24 | | | |
| N _ItemNr_ | 160 | | | |
| Observations | 3203 | | | |
| Marginal R^2^ / Conditional R^2^ | 0.020 / 0.080 | | | |
| Amplitude ~ Switch * Partner * Half + (1 \| Subject) + (1 + Switch \| ItemNr); ** p<0.05   ** p<0.01   *** p<0.001* | | | | |

## Experiment 2, LPC, n=24

| ***Table S.18 Linear mixed-effects model, Experiment 2, n=24*** | | | | |
| --- | --- | --- | --- | --- |
|  | **Amplitude 500-900 ms, Central-Parietal sites** | | | |
| *Parameters* | *Estimate* | *SE* | *t* | *p* |
| Intercept | 2.76 ^***^ | 0.34 | 8.11 | **<0.001** |
| Switch | 3.31 ^***^ | 0.5 | 6.59 | **<0.001** |
| Partner | 1.29 ^***^ | 0.29 | 4.38 | **<0.001** |
| Half | -0.66 ^*^ | 0.29 | -2.25 | **0.024** |
| Switch:Partner | 1.25 ^*^ | 0.59 | 2.11 | **0.035** |
| Switch:Half | -0.29 | 0.58 | -0.5 | 0.62 |
| Partner:Half | -1.85 | 1.32 | -1.4 | 0.161 |
| Switch:Partner:Half | 0.3 | 1.95 | 0.16 | 0.876 |
| N _Subject_ | 24 | | | |
| N _ItemNr_ | 160 | | | |
| Observations | 3203 | | | |
| Marginal R^2^ / Conditional R^2^ | 0.048 / 0.111 | | | |
| Amplitude ~ Switch * Partner * Half + (1 \| Subject) + (1 + Switch \| ItemNr);**p<0.05   ** p<0.01   *** p<0.001* | | | | |

# Outcomes of statistical models excluding Spanish-dominant participants

## Experiment 1, Early Frontal Positivity

| ***Table S.19 Linear mixed-effects model, English-dominant participants Experiment 1, n=14*** | | | | |
| --- | --- | --- | --- | --- |
|  | **Amplitude 200-300 ms, Fronto-central sites** | | | |
| *Parameters* | *Estimate* | *SE* | *t* | *p* |
| Intercept | 2.86 ^***^ | 0.45 | 6.33 | **<0.001** |
| Switch | 2.28 ^***^ | 0.64 | 3.56 | **<0.001** |
| Half | -0.44 | 0.42 | -1.04 | 0.300 |
| Switch:Half | 0.42 | 0.84 | 0.50 | 0.616 |
| N _Subject_ | 14 | | | |
| N _ItemNr_ | 160 | | | |
| Observations | 1651 | | | |
| Marginal R^2^ / Conditional R^2^ | 0.017 / 0.065 | | | |
| Amplitude ~ Switch * Half + (1 + Switch \| Subject) + (1 \| ItemNr) ** p<0.05   ** p<0.01   *** p<0.001* | | | | |

## Experiment 1, LAN

| ***Table S.20 Linear mixed-effects model, Eng Dominant, Experiment 1, n=14*** | | | | |
| --- | --- | --- | --- | --- |
|  | **Amplitude 300-500 ms, Left Frontal sites** | | | |
| *Parameters* | *Estimate* | *SE* | *t* | *p* |
| Intercept | -0.30 | 0.27 | -1.12 | 0.283 |
| Switch | 1.36 ^***^ | 0.37 | 3.65 | **<0.001** |
| Half | -0.45 | 0.38 | -1.22 | 0.223 |
| Switch:Half | 0.72 | 0.74 | 0.97 | 0.332 |
| N _Subject_ | 14 | | | |
| N _ItemNr_ | 160 | | | |
| Observations | 1651 | | | |
| Marginal R^2^ / Conditional R^2^ | 0.009 / 0.028 | | | |
| Amplitude ~ Switch * Half + (1 \| Subject) + (1 \| ItemNr); ** p<0.05   ** p<0.01   *** p<0.001* | | | | |

## Experiment 1, N400

| ***Table S.21 Linear mixed-effects model, Eng Dominant, Experiment 1, n=14*** | | | | |
| --- | --- | --- | --- | --- |
|  | **Amplitude 300-500 ms, Central sites** | | | |
| *Parameters* | *Estimate* | *SE* | *t* | *p* |
| Intercept | 0.17 | 0.29 | 0.56 | 0.574 |
| Switch | 1.48 ^***^ | 0.39 | 3.81 | **<0.001** |
| Half | -0.62 | 0.39 | -1.59 | 0.113 |
| Switch:Half | 0.91 | 0.78 | 1.17 | 0.242 |
| N _Subject_ | 14 | | | |
| N _ItemNr_ | 160 | | | |
| Observations | 1651 | | | |
| Marginal R^2^ / Conditional R^2^ | 0.011 / 0.034 | | | |
| Amplitude ~ Switch * Half + (1 \| Subject) + (1 \| ItemNr); ** p<0.05   ** p<0.01   *** p<0.001* | | | | |

## Experiment 1, LPC English Dominant

| ***Table S.22 Linear mixed-effects model, English Dominant, Experiment 1, n=14*** | | | | |
| --- | --- | --- | --- | --- |
|  | **Amplitude 500-900 ms, Central-Parietal sites** | | | |
| *Parameters* | *Estimate* | *SE* | *t* | *p* |
| Intercept | 1.65 ^***^ | 0.37 | 4.48 | **<0.001** |
| Switch | 3.42 ^***^ | 0.50 | 6.80 | **<0.001** |
| Half | -1.21 ^**^ | 0.38 | -3.17 | **0.002** |
| Switch:Half | -0.08 | 0.77 | -0.10 | 0.917 |
| N _Subject_ | 14 | | | |
| N _ItemNr_ | 160 | | | |
| Observations | 1651 | | | |
| Marginal R^2^ / Conditional R^2^ | 0.050 / 0.102 | | | |
| Amplitude ~ Switch * Half + (1 + Switch \| Subject) + (1 \| ItemNr);** p<0.05   ** p<0.01   *** p<0.001* | | | | |

## Experiment 2, Early Frontal Positivity

| ***Table S.23 Linear mixed-effects model, Experiment 2, English dominant, n=31*** | | | | |
| --- | --- | --- | --- | --- |
|  | **Amplitude 200-300 ms, Fronto-central sites** | | | |
| *Parameters* | *Estimate* | *SE* | *t* | *p* |
| Intercept | 3.68 ^***^ | 0.37 | 9.96 | **<0.001** |
| Switch | 1.84 ^***^ | 0.33 | 5.63 | **<0.001** |
| Partner | -0.02 | 0.27 | -0.09 | 0.932 |
| Half | -0.56 ^*^ | 0.27 | -2.06 | **0.039** |
| Switch:Partner | -0.17 | 0.55 | -0.31 | 0.755 |
| Switch:Half | -0.07 | 0.55 | -0.13 | 0.894 |
| Partner:Half | -1.00 | 1.44 | -0.7 | 0.486 |
| Switch:Partner:Half | -2.71 ^*^ | 1.32 | -2.06 | **0.04** |
| N _Subject_ | 31 | | | |
| N _ItemNr_ | 160 | | | |
| Observations | 4097 | | | |
| Marginal R^2^ / Conditional R^2^ | 0.014 / 0.074 | | | |
| Amplitude ~ Switch * Partner * Half + (1 + Switch \| Subject) + (1 \| ItemNr); ** p<0.05   ** p<0.01   *** p<0.001* | | | | |

## Experiment 2, LPC

| ***Table S.24 Linear mixed-effects model, English dominant Experiment 2, n=31*** | | | | |
| --- | --- | --- | --- | --- |
|  | **Amplitude 500-900 ms, Central-Parietal sites** | | | |
| *Parameters* | *Estimate* | *SE* | *t* | *p* |
| Intercept | 2.52 ^***^ | 0.34 | 7.33 | **<0.001** |
| Switch | 3.22 ^***^ | 0.45 | 7.23 | **<0.001** |
| Partner | 1.00 ^***^ | 0.25 | 3.96 | **<0.001** |
| Half | -0.69 ^**^ | 0.25 | -2.77 | **0.006** |
| Switch:Partner | 0.77 | 0.5 | 1.53 | 0.126 |
| Switch:Half | -1.13 ^*^ | 0.5 | -2.25 | **0.025** |
| Partner:Half | -1.84 | 1.34 | -1.37 | 0.17 |
| Switch:Partner:Half | 0.58 | 1.71 | 0.34 | 0.733 |
| N _Subject_ | 31 | | | |
| N _ItemNr_ | 160 | | | |
| Observations | 4097 | | | |
| Marginal R^2^ / Conditional R^2^ | 0.046 / 0.123 | | | |
| Amplitude ~ Switch * Partner * Half + (1 + Switch \| Subject) + (1 + Switch \| ItemNr); ** p<0.05   ** p<0.01   *** p<0.001* | | | | |

# Correlations with Autism Quotient

## Correlation between AQ and “yes” responses to “Did your partner understand the question?” in switch condition with monolingual partner

Pearson's product-moment correlation: 0.23

*t*(31) = 1.32, *p* > 0-1

## Correlation between AQ and LPC switch effect

Switch effect is calculated as the difference in amplitude (500-900 ms, central-parietal electrodes) for Switch minus No-switch trials in the monolingual partner condition

### All participants

Pearson's product-moment correlation: -0.29

*t*(31) = -1.69, *p* > 0.1

### Only those who responded “yes” < 37.5% of the time (n=21)

Pearson's product-moment correlation: -0.28

*t*(19) = -1.29, *p* > 0.2
